# The Venereal Report Appears

**Published:** 1921-03-05

**Authors:** 


### THE VENEREAL REPORT APPEARS.

THE recently published report on Prevention of Venereal Disease* drawn up by the Special Committee of the Birth-rate Commission, deals with a gigantic problem in a manner which should receive the approval of a scientific profession, and at the same time make the situation as it really is considerably clearer to the moralist. As comprehensively as possible, with so thorny a subject, the Committee have put forward all the facts. It is not implied that discussion thereon is finished, but the report forms a thoroughly sound basis for constructive work.

The Committee first deals with the medical aspects of the question. Venereal, in common with other infectious diseases, allows of two methods in preventing its spread. First, we may prevent those conditions under which contagion occurs; second, the infective organisms may be, by disinfection, destroyed before they have penetrated the tissues of the body. Obviously, the first procedure is the best, and we see it successfully carried out in a host of well-known parasitic and bacterial diseases. But with venereal disease, as the report plainly states, although it is to be realised that chastity is the best safeguard, yet it must be once and for all admitted that a large number of persons, male and female, do not respond to moral appeals, upon which the success of this method depends. Therefore the Committee from practical considerations favour immediate self-disinfection, at the same time admitting its liability to failure in circumstances of carelessness, ignorance, etc.

So far, then, a valuable, if not novel, decision has been expressed, but in dealing with the scourge in its wider aspects the later findings are important. How and in what form is public instruction to be provided? In the first place, it is recommended that health authorities should uphold the value of chastity as being the one and only sure means of prevention, but should urge, though not unduly, the efficacy of self-disinfection. This should be put forward simply as a method of first-aid in preventing possible danger, and to be followed by immediate resort to medical advice. Since a person should be fully able to carry out the necessary process, official provision of special facilities and ablution centres is not advocated; this attitude is held to be justified by the fact that the person's risk has been incurred as a result of his own misconduct.

But coincident with this individual method there are, of course, certain practical essentials. Legal prohibition of sale of disinfectants by a chemist will have to be withdrawn, though he still must not advertise or recommend non-approved substances. Again, these disinfectants will preferably be accompanied by such instructions as the Ministry of Health may detail, and, meanwhile, the prohibition must still apply to all unauthorised persons. There are other minor points; and, broadly speaking, these things sound thoroughly practicable. When, later, the report proceeds to deal with the moral side, and we read, among other things, that no legislation can be recommended making it obligatory for all parties contemplating marriage to produce health certificates, we recognise that divergence of opinion will occur (see p. 519). On the other hand, the suggested phases in a public appeal and their relative importance are less open to criticism. First is placed chastity as a moral obligation and the only sure preventive. Secondly, there is the moral obligation to consult a doctor. Thirdly comes the serious risk involved demanding an appeal to family affection, patriotism and humanity. And, last in order of prominence, instruction in self-disinfection is urged. The whole matter is well and simply put forward, and we heartily endorse the President's strong appeal that the two societies in opposition should now come together and find a *modus vivendi* based upon the report, for it is hard to reconcile with an apparent disunion among known authorities with efficient solution of so grave and urgent a problem.

* To be obtained from the Secretary, Dr. James Marchant, C.B.E., 60 Gower Street, W.C., and published by Williams and Norgate, London.

